# Microbiota and Malodor—Etiology and Management

**DOI:** 10.3390/ijms21082886

**Published:** 2020-04-20

**Authors:** Izabella Mogilnicka, Pawel Bogucki, Marcin Ufnal

**Affiliations:** 1 Department of Experimental Physiology and Pathophysiology, Laboratory of the Centre for Preclinical Research, Medical University of Warsaw, 02-091 Warsaw, Poland; izabella.mogilnicka@gmail.com; 2 Bristol Dermatology Centre, University Hospitals Bristol, Bristol BS2 8HW, UK; boguckipawel@yahoo.co.uk

**Keywords:** gut microbiota, host interactions, symbiosis, dysbiosis, halitosis, malodor

## Abstract

Accumulating evidence indicates that microbiota plays a critical role in physiological processes in humans. However, it might also contribute to body malodor by producing numerous odorous molecules such as ammonia, volatile sulfur compounds or trimethylamine. Although malodor is commonly overlooked by physicians, it constitutes a major problem for many otherwise healthy people. Thus, this review aims to investigate most common causes of malodor and describe potential therapeutic options. We searched PUBMED and Google Scholar databases to identify the clinical and pre-clinical studies on bad body smell, malodor, halitosis and microbiota. Unpleasant smell might originate from the mouth, skin, urine or reproductive fluids and is usually caused by odorants that are produced by resident bacterial flora. The accumulation of odorous compounds might result from diet, specific composition of microbiota, as well as compromised function of the liver, intestines and kidneys. Evidence-based guidelines for management of body malodor are lacking and no universal treatment exists. However, the alleviation of the symptoms may be achieved by controlling the diet and physical elimination of bacteria and/or accumulated odorants.

## 1. Introduction

Patient’s complaints of unpleasant body smell or breath odor require ruling out life-threatening diseases such as diabetes mellitus or liver failure in the first place. Next, other less serious conditions such as sinusitis, gastroesophageal reflux or dental problems need to be investigated. The exclusion of the most common conditions associated with body odor will likely result in advising the patient to draw more attention to personal hygiene. Though, frequent showers and teeth brushing may not solve the problem.

Rising number of support groups for patients with malodor indicates that this issue is an important public health problem. Unfortunately, conditions that are characterized by an unpleasant smell but not associated with other significant health problems are not covered by evidence-based medical guidelines or curriculums at most of medical schools, and so physicians are inadequately trained on that matter.

It needs to be stressed that the chronic malodor might lead to serious psychological problems. Patients with bad smell might adopt various behaviors minimizing contact with their surroundings, which can result in anxiety, decreased self-esteem and low quality of life due to social difficulties such as withdrawing from personal contacts or avoiding intimacy, which lowers a chance of finding life-partner [1]. McKeown reported that as much as 75% of patients of the clinic specializing in treating halitosis sought medical help due to social consequences of their condition [2].

The aim of this review is to summarize the current knowledge on origins and management of body odor resulting from other than health- or life-threatening diseases. This article focuses on body odors that are caused by bacteria-derived products which are the most common reason for unpleasant smell.

## 2. Origins of Body Odor Associated with Bacterial Metabolites

**Figure 1:**
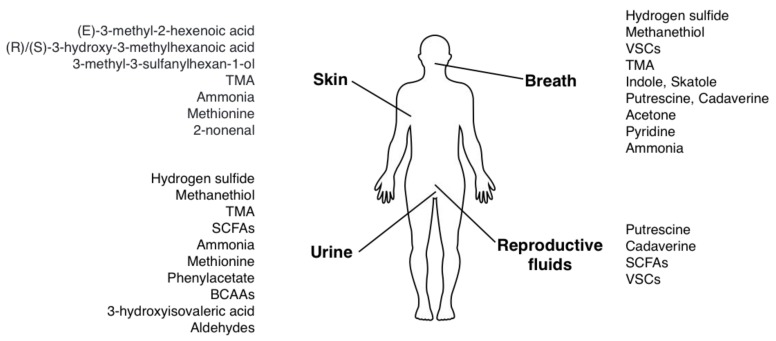
Origins of body odor associated with bacterial metabolites.

The body can emit odorous substances (odorants) with breath, saliva, sweat (skin), urine or reproductive organs fluids. The major odorants are small, volatile compounds that may either be produced in situ (skin, oral cavity) or be carried by blood from the gut, which is a major site of bacterial metabolism. Main bacterial odorants and their emission sites are presented in Figure 1.

It needs to be highlighted that most of these molecules are present under physiological conditions and some of them play important biological functions. However, an excessive accumulation of these compounds is associated with unpleasant smell.

Malodor that is associated with accumulation of bacterial metabolites in body fluids might result from one or more of the following causes:

1. Diet. Diet contains direct or indirect odorants (i.e., substrates for the production of odorants by bacteria).
2. Composition and metabolic activity of bacteria in the gut, skin or mucosa. The production of specific odorant is often limited to specific genera or strains of bacteria.
3. Gut function. Intestinal transit time and gut-blood barrier permeability affect penetration of bacterial metabolites and their precursors from the gut lumen to the bloodstream. Recently the concept of a leaky-gut has attracted a lot of attention, as several studies show that numerous diseases may affect intestinal barrier function and increase the penetration of bacterial metabolites to the circulation.
4. Liver function. Liver metabolizes most of gut-derived bacterial metabolites, reducing the “odorant potential” of the metabolites (e.g., very odorant trimethylamine is transformed to almost neutral trimethylamine oxide; hydrogen sulfide is transformed to numerous sulfur compounds, etc.).
5. Kidney function. Kidney excretion is crucial for the elimination of both bacterial metabolites and substrates for their production from blood.

## 3. Breath and Saliva

Halitosis (fetor oris or bad breath) is a condition characterized by oral malodor of either intra-oral or extra-oral origin [3,4,5,6]. About 20–50% of adult or adolescent individuals suffer from oral malodor worldwide. A more precise number was recently reported in a systematic review by Silva et al. –31.8% (95% CI 24.6–39.0%) [7].

In a vast majority (80–90%) of patients with persistent bad breath the reason for that complaint is of oral origin with tongue coating and periodontal disease being the most prevalent [5,8]. Among extra-oral (non-oral) causes of halitosis diabetic ketoacidosis [9,10], congenital metabolic diseases [11,12,13], gastrointestinal [14,15] or respiratory [16,17] conditions should be recognized. It is also worth mentioning that although halitosis might be an indication of some serious medical conditions, consumption of several food products such as onion or garlic as well as smoking might also result in bad breath.

Predominant substances present in breath of individuals suffering from halitosis are volatile sulfur compounds (VSCs) such as hydrogen sulfide (H_2_S) [18,19,20,21,22], ethanethiol, S-ethyl thioacetate, diethyl disulfide, dimethyl sulfide ((CH_3_)_2_S) [20,21,23,24,25] and methanethiol (CH_3_SH or methyl mercaptan) [20,26]. Several Gram–negative bacteria e.g., *Bacteroides forsythus*, *Porphyromonas gingivalis*, *Actinobacillus actinomycetemcomitans* and *Prevotella intermedia* [27] have been associated with the production of volatile sulfur compounds from sulfur-containing substrates that are present in food or saliva. However, no direct association has been recognized and complex microbial interactions are suspected. Other odorous compounds include indole [28,29], skatole [28,29,30], putrescine [30,31], cadaverine [32], pyridine [33], ammonia [34,35,36,37], trimethylamine [38,39,40], acetone [9,36,41,42,43] and products of metabolism of methionine [11,12,13]. All odorants described in following paragraphs have been summarized in Table 1 and Table 2 along with conditions associated with their emission.

Odorous substances in halitosis are discussed below:

### 3.1. H_2_S–Smell of Rotten Eggs

At low concentrations hydrogen sulfide (H_2_S) is an important biological mediator similarly to other gaseous transmitters such as nitric oxide or carbon monoxide [100]. However, at higher concentrations H_2_S is mostly known for its toxic effects and specific foul smell, often compared to the odor of rotten eggs [44]. Even small concentrations of H_2_S cause significant smell, because of its very high odor index and very low detection threshold (around 4 nmol/L = 96 ppb) [20,23].

It is commonly known that anaerobic bacteria present in the oral cavity (subgingival microbiota–flora collected from periodontal pockets) might degrade sulfur-containing aminoacids such as l-cysteine to odorous volatile sulfur compounds with H_2_S being an important contributor to offensive breath odor. Decades ago Persson and collaborators have reported the production of hydrogen sulfide by *Treponema denticola* and *Bacteroides intermedius*, as well as other genera e.g., *Peptostreptococcus*, *Eubacterium* and *Fusobacterium* [18]. Many years later Washio et al. [45] aimed to identify H_2_S–producing bacteria in a tongue biofilm of patients suffering from halitosis using gas chromatography and sulfide monitor to assess H_2_S levels. *Veillonella* spp., *Actinomyces* spp. and *Prevotella* spp. have been found to be dominant in patients suffering from oral malodor. On the other hand, Takeshita et al. [19] described *Neisseria* spp., *Fusobacterium* spp. and *Porphyromonas* spp. to be the most prevalent in the oral cavity of patients with high concentration of H_2_S in breath, while *Veilonella* spp. and *Prevotella* spp. were dominant in patients exhaling mostly CH_3_SH.

It is worth remarking that in certain food products, substrates for the production of H_2_S by bacterial reductases might occur. For example onion, garlic, wine, cabbage, broccoli, mushrooms, nuts, potatoes and dried fruits contain sulfides and sulfites (used as antibacterial and antioxidizing agents) [101,102,103].

### 3.2. Methanethiol (CH_3_SH/MT/MeSH) = Methyl Mercaptan–Putrid, Musty Smell

Another molecule detected in breath of patients suffering from halitosis is methyl mercaptan. Studies show that CH_3_SH is a predominant causative factor of intra-oral halitosis [20,46]. The smell of MT is often described as putrid, musty and can be compared to barnyard odor. In fact, malodor of patients with halitosis is usually more similar to that than to the odor of rotten eggs (characteristic for H_2_S) [23]. The threshold of objectionability is 0.5 nM (12 ppb) [23]. Similarly to H_2_S, MT cannot be detected in the mouth air of patients with extra-oral (blood-borne) halitosis, where other VSCs play a crucial role [20,23].

Aiming to find a source of methyl mercaptan in patients with halitosis, Takeshita and collaborators [19] investigated bacterial communities in oral cavities of patients with odorous breath while using molecular techniques. The researchers reported high prevalence of *Prevotella, Veillonella, Atopobium, Megasphaera*, and *Selenomonas* in saliva of these individuals and suspect that these species are involved in the production of CH_3_SH. Yaegaki et al. [26] measured the amount of MT in mouth air of patients with oral malodor and reported its significantly increased concentration in individuals suffering from periodontal disease. Yaegaki also concluded that tongue coating has an important role in the production of MT; however Tangerman et al. [23] proved that the degree of coating is not as important as its presence in general.

### 3.3. Other Volatile Sulfur Compounds (VSCs)–Sweet, Musty Smell of Cooked Onion

Other volatile sulfur compounds that are detected in breath and saliva of patients with halitosis include ethanethiol, S-ethyl thioacetate, diethyl disulfide and dimethyl sulfide. These compounds are associated with distinctive, sweet, musty smell of vegetables, often being described as similar to the odor of cooked onion [20,23,104].

Tangerman et al. [23] performed gas chromatography on mouth and nose breath samples of patients with halitosis and concluded that dimethyl sulfide (DMS, CH_3_SCH_3_) is the main contributor to blood-borne (extra-oral) malodor. In contrast, the concentration of DMS in mouth and nose breath of individuals with intra-oral halitosis was in the normal range and did not reach odorous threshold. Possible reason for the presence of DMS in blood is some metabolic disorder that needs to be further investigated. Other causes reported in literature include hepatic failure [94], hypermethioninemia (an inherited methylation disorder primarily associated with elevated levels of methionine) [11,12] and therapeutic intake of dimethyl sulfoxide [105] or cysteamine [106].

### 3.4. Trimethylamine–Fishy Smell

Trimethylamine (TMA) is a volatile, aliphatic tertiary amine known for its characteristic odor of rotten fish and toxic effects at high concentrations [107,108]. It is formed from excess choline and other TMA-containing dietary nutrients by gut bacteria. It has been shown that many symbiotic bacteria residing in the gut e.g., *Anaerococcus, Providencia, Edwardsiella, Clostridium, Collinsella, Desulfovibrio, Lactobacillus* and *Proteus* produce TMA by metabolizing diet-derived TMA-containing substances [51,109,110,111,112,113].

After being absorbed from the intestines, TMA is oxidized by the liver to almost odorless trimethylamine oxide (TMAO). In trimethylaminuria (TMAu or “fish odor syndrome”) TMA is accumulated and excreted to the body fluids, because of deficiency of flavin-containing monooxygenase 3 (FMO3) [49,50], a liver enzyme oxidizing TMA.

There are two major types of TMAu and a few transient forms of this condition. While primary (inherited) TMAu is caused by one of almost 20 detected genetic mutations in FMO3 gene, leading to functional deficiency of FMO3 [50,114], secondary (acquired) type has been reported in patients with severe liver [38,115,116] or renal [92] disease. Moreover, a transient form in children (related to choline-containing food supplement intake) and in women (associated with menstruation) has been described in literature [39,117,118]. That metabolic block results in substrate overload and distinctive body malodor, which can be detected in multiple body sites including breath.

Even though the process of diagnosis is quite straightforward, as it is mostly based on the biochemical screening of urine samples (quantitation of TMA and TMAO) and/or FMO3 mutational analysis, TMAu is still an under-recognized disease and patients sometimes remain undiagnosed for decades [49,88,119]. TMAu should always be considered as a possible reason for bad breath smell.

### 3.5. Indole and Skatole–Smell of Feces

Indoles represent a group of microbiota-derived compounds produced from tryptophan, an essential amino acid and the precursor for endogenous synthesis of tryptamine, serotonin and melatonin [120].

Indoles that have been suspected of contributing to bad breath include indole and skatole, which are produced by intra-oral anaerobic Gram-negative bacteria such as *Porphyromonas intermedia, Fusobacterium nucleatum* and *Porphyromonas gingivalis* [28].

Their smell can be equated to that of fecal matter and of these two compounds skatole [30] has a stronger odor. In comparison to volatile sulfur compounds, indole and skatole are far less volatile and as so, their contribution to halitosis is rather minimal. However, some individuals with halitosis might have undetectable concentrations of VSCs in breath along with high levels of indoles and in such a group of patients popular tools used to determine breath odor (e.g., Halitometer that detects VSCs) might be insufficient and delay diagnosis.

### 3.6. Putrescine and Cadaverine–Smell of Rotten Meat or Fish

Putrescine and cadaverine, diamines that have been suspected to contribute to the foul smell of breath for a few decades, are both associated with putrefaction of food by bacteria present in the dental plaque [57]. They are produced in saliva by amino-acid decarboxylation (of lysine and ornithine respectively) or transamination [32,57,121,122].

The odor of putrescine is often compared to that of spoiled fish or rotten meat while the smell of cadaverine besides aforementioned might also remind the smell of urine or semen [58]. Goldberg and colleagues [32] reported strong correlation between oral malodor and presence of cadaverine in saliva.

### 3.7. Acetone–Fruity Smell

Acetone is a three-carbon, volatile ketone that is derived from acetoacetate (through decarboxylation or enzymatic conversion) [62]. Its fruity smell (often compared to that of rotten apples) has been associated with uncontrolled diabetes mellitus (DM) for a long time [60,61]. However, only recent advances in analytical methods have allowed for the identification of specific compounds in the exhaled air of patients with DM. High concentrations of breath acetone have been linked with diabetic ketoacidosis [62]. It also increases with fasting, high-fat or ketogenic diet (which has become popular among those who claim its positive pro-cognitive effect) [123].

### 3.8. Pyridine–Fishy, Sweaty Smell

Pyridine is an aromatic nitrogen-containing, volatile compound that has a fishy, sweaty odor [63] and might contribute to halitosis. Although very little research have been done on the role of this substance in oral malodor, pyridine and its analogues (2-, 3- and 4-methylpyridines) have been reported in the incubated saliva of patients with moderate and severe periodontal disease [64]. In contrast, these molecules have been absent in samples from patients with good oral health [33].

### 3.9. Ammonia–Urine-Like Smell

Ammonia (NH_3_) is present in a form of ammonium ion (NH_4_^+^) in all body fluids, but its high concentrations are toxic, hence it is precisely regulated by the urea cycle [124]. Its levels can be measured in breath, saliva, blood, urine or sweat.

With its urine-like, fetid smell, ammonia plays an important role as a biomarker in liver [93,125,126] and kidney [35,37,127] diseases as well as in halitosis [71]. Amano et al. [71] reported decrease of ammonia level in breath after removal of tongue coating and dental plaque, which might suggest that some microorganisms present in oral cavity are responsible for the production of NH_3_ in intra-oral halitosis. Chen and collaborators [128] suspect that mouth-exhaled NH_3_ comes from hydrolysis of oral fluid urea which is performed by bacterial urease and that in certain pH in oral cavity fluid ammonia turns into gaseous form.

In patients suffering from end-stage renal disease (ESRD) uremic odor of breath is caused by high concentrations of urea in saliva, which is broken down to ammonia [91]. In a study by Kho et al. as much as 34.1% of subjects with ESRD complained of uremic fetor [35]. These results are further supported by Anuradha et al. [34].

Liver failure was historically associated with urine-like fetor hepaticus (hepatic breath). Shimamoto et al. [93] compared levels of blood and breath ammonia in patients with and without cirrhosis. Patients suffering from hepatic encephalopathy had significantly higher levels of breath ammonia than controls. Also, both breath and blood ammonia decreased with treatment of hyperammonemia. Supporting evidence has been reported by Spacek and colleagues [126].

Gut bacteria are important contributors to formation of ammonia in mammals. Bacteria (mostly gram-negative *Enterobacteriaceae*) inhabiting the GI tract produce urease that hydrolyzes urea into carbon dioxide and ammonia [129]. Other bacterial strains e.g., *E. coli* and *Salmonella enterica* are able to form ammonia from cysteine by cysteine desulfhydrase [130]. Moreover, *E. coli* can also reduce nitrates to ammonia [131]. Gut-derived ammonia is then either utilized by the gut bacteria for protein resynthesis, absorbed through GBB (gut-blood barrier) into circulation or excreted with feces [129,132]. In normal conditions ammonia produced in the gut is metabolized in the liver. However, in the state of liver failure it cannot be converted into non-toxic derivatives such as urea or glutamine.

Because NH_3_ is also formed during protein catabolism [128], it can be assumed that a diet rich in proteins might increase its blood levels and result in greater amount of mouth-exhaled ammonia. Indeed, research shows that the ingestion of proteins results in increased serum and saliva urea which in result increases concentration of breath ammonia [133]. This needs to be recognized, especially since many popular, unhealthy fad diets are based on high protein consumption [134,135].

## 4. Urine

Urine components are affected by body metabolism but also by consumed food and drinks. Therefore, it needs to be stressed that not every odor noted in the urine should be recognized as alarming. For example, shortly after ingestion of asparagus urine might have a distinct sulfurous smell (reminiscing cooked cabbage) in some individuals. Although exact molecules that are responsible for that odor have not been unambiguously identified, several VSCs like methanethiol or dimethyl sulfide are suspected [48].

The identification of specific odorous compounds in urine might be useful in the diagnosis of conditions like phenylketonuria [77], hypermethioninemia [12] or maple syrup urine disease [79]. It is important to mention that multiple odorous substances are produced by gut flora and they can appear in urine after absorption from intestine to the circulation. Several substances affecting smell of the urine will be covered below.

### 4.1. H_2_S–Smell of Rotten Eggs

Among patients with urinary incontinence (UI, involuntary leakage of urine that significantly affects quality of life [82]) unpleasant smell is one of the major complaints and an important cause for social embarrassment. In a research conducted by Pandey et al. [82] major volatile odorants in urine and in absorbent incontinence pads were studied. Along with other molecules such as methanethiol and aldehydes, hydrogen sulfide was detected above the odor threshold. Also, in urinary tract infections *E. coli* is a common producer of odorous hydrogen sulfide [136].

### 4.2. Methanethiol–Putrid, Musty Smell

Gaseous methanethiol in the urine of a healthy person is below the detection threshold [20]. Elevated levels have been found in patients treated with cysteamine [106], in hepatic methionine adenosyltransferase deficiency [11] and in UI [82]. Major contributors to methanethiol production are gut bacteria such as *E. coli*, *Citrobacter* and *Proteus* [137]. Methanethiol is absorbed from the intestine, enters circulation and can be then excreted with urine.

### 4.3. Trimethylamine–Fishy Smell

As aforementioned, in trimethylaminuria an excess of trimethylamine that cannot be oxidized to odorless TMAO, is secreted to multiple body fluids, including urine. It needs to be stressed that some patients only have intermittent TMAu which makes establishing the right diagnosis more difficult, as the urine tests can come out negative during the period when odor is not prominent and have to be repeated [49]. For example, menstruating women should be tested during or right before their menstruation in order to maximize the chances of detecting TMAu, as it has been reported that TMA excretion increases during that time [117].

Miller et al. [52] reported on a child with transient trimethylaminuria associated with food protein-induced enterocolitis syndrome with massive urinary TMA excretion during acute enterocolitis. Mitchell et al. [38] described specific odor in patients with secondary TMAu in liver disease. In their study more than 25% of enrolled patients excreted amounts of TMA that were above the odor threshold. Another source of urinary TMA could be gut flora, mainly *Anaerococcus, Providencia, Edwardsiella, Clostridium, Collinsella, Desulfovibrio, Lactobacillus* and *Proteus* as described above.

### 4.4. Short Chain Fatty Acids–Cheesy Smell

Isovaleric acidemia (IA) is often associated with specific cheesy body odor of “sweaty feet” or human vomit [54] which can be noted during metabolic crisis. This disease is a result of deficiency of isovaleryl-CoA dehydrogenase (IVD) leading to abnormal metabolism of leucine. The pathognomonic substance that can be detected in urine of patients with IA is isovalerylglycine [55,97].

### 4.5. Ammonia–Urine-Like Smell

Ammonia was one of the first molecules that were thought to cause malodor around patients with urine incontinence [72]. Bacterial ureases (mainly *Escherichia coli*, *Proteus mirabilis,* and *Enterococcus faecalis*) would break down urea to foully smelling ammonia, according to that conception [138]. However, this assumption was challenged by Pandey et al. [82].

### 4.6. Methionine and its Metabolites–Smell of Rancid Butter or Boiled Cabbage

Methionine adenosyltransferase (MAT) I/III deficiency is an inherited error of methionine metabolism which is mostly detected in newborn screening. It is caused by mutations in the MAT1A gene resulting in the accumulation of methionine and its metabolites [13,96]. In fact, according to Barić et al. [12] it is the most common cause of persistent isolated hypermethioninemia.

### 4.7. Phenylacetate–Musty, Mousy Smell

Another cause for distinctive, mousy smell of urine is phenylketonuria (PKU). Being a metabolic disease, it is beyond the focus of this review. More information on this issue can be found elsewhere [77].

### 4.8. Branched-Chain Amino Acids (Leucine, Isoleucine and Valine) and their Ketoacids–Smell of Caramelized Sugar or Maple Syrup

Maple syrup urine disease (MSUD, leucinosis) is another error of metabolism with distinctive urine smell as one of the symptoms. Further details can be found elsewhere [78,79].

### 4.9. 3-Hydroxyisovaleric Acid–Smell of Male Cat Urine

Among other symptoms of 3-methylcrotonyl-CoA carboxylase (3-MCC) deficiency, specific smell of urine has been reported by some researchers. Other authors have discussed this condition in more detail [80,81].

### 4.10. Aldehydes (Acetaldehyde, Butylaldehyde, Isovaleraldehyde)–Urine-Like Smell

A few odorous aldehydes have been detected in urine samples and incontinence hygienic pads from patients with urinary incontinence in aforementioned study by Pandey and collaborators [82]. Acetaldehyde, butylaldehyde, isovaleraldehyde (that have foul, urine-like smell) were reported in concentrations above detection threshold and it can be assumed that they effectively contribute to odor intensity in patients with UI. The origin of urinary aldehydes must be at least partially the gut and its microbiota.

## 5. Sweat and Skin

Volatile odorous molecules that are emitted from skin surface are mostly derived from sweat, which is a product of sweat glands’ secretion. Three main types of human sweat glands should be distinguished: apocrine, eccrine and apoeccrine (mixed). Eccrine glands produce large amounts of sweat containing mostly water and electrolytes. They are distributed over almost the entire body surface. On the other hand, apocrine glands are located mostly in the axillary, perineal, genital regions and around the nipples. They become active after puberty and secrete less sweat than eccrine glands. Apocrine sweat is odorless and it only becomes odorous after breakdown by microorganisms inhabiting skin surface such as *Micrococcaceae*, aerobic diphtheroids and *Propionibacteria* [139,140,141]. Apocrine glands secrete sweat similar to this produced by eccrine glands, but their secretion rate is higher, and they are particularly present in axillary region. These features make apocrine glands significant contributors to axillary sweating [139].

Changes in hormonal balance, consumed food and metabolic shifts might have an impact on both quantitative and qualitative composition of sweat. Any shifts in skin microbiota as well as bacterial infections might change the composition of sweat, often producing distinctive odor, given that human organism remains in symbiosis with several microbial species that are able to transform chemical compounds of sweat. For example, in streptococcal intertrigo a distinctive foul smell of patients’ skin has been reported [142].

Additionally, numerous metabolic disorders are characterized by a variety of odors that are emitted from the skin. Some of these conditions include phenylketonuria [89], methionine malabsorption syndrome [13], hypermethioninemia [76], or TMAu [53].

Substances that are known to contribute to odor emitted from the skin will be discussed below.

### 5.1. (E)-3-Methyl-2-Hexenoic acid (E3M2H), (R)/(S)-3-Hydroxy-3-Methylhexanoic Acid ((R)/(S)-HMHA) and 3-Methyl-3-Sulfanylhexan-1-ol ((R)/(S)-MSH)–Rancid, Cheesy Smell of Sweat

Bromhidrosis, which is also known as osmidrosis or malodorous sweating, is a distressing condition that is characterized by offensive body odor, noticeable especially in axillary, genital or feet area. All three types of sweat glands play a role in pathogenesis of this disease. Excessive sweating followed by decomposition of sweat constituents by bacteria results in unpleasant smell of sweat. Apocrine bromhidrosis which in contrary to eccrine bromhidrosis develops after puberty, is the most common form of this condition [139]. Bacteria break down apocrine sweat into numerous volatiles molecules such as ammonia and short chain fatty acids e.g., (E)-3-methyl-2-hexenoic acid (E3M2H) [66] which is a C_7_ branched and unsaturated acid. It has been reported to have a very strong, pungent odor [67]. Natsch et al. [65] reported that odorous E3M2H along with its hydrated analogue, (R)/(S)-3-hydroxy-3-methylhexanoic acid ((R)/(S)-HMHA) are released from glutamine conjugates (that are present in axilla secretions) by a specific zinc-dependent *N*-α-acyl-glutamine aminoacylase (N-AGA) from commensal *Corynebacterum* species that reside on the skin of axilla. HMHA (characterized by a rancid, cheesy smell) has been reported the most abundant. (S)-isomer of 3-methyl-3-sulfanylhexan-1-ol ((R)/(S)-MSH) with its oniony, clary sage-like smell is another particle that is responsible for axillary malodor [68,69,70]. Further, multiple factors have been associated with eccrine bromhidrosis. These include ingestion of certain food products such as garlic or onion, bacterial degradation of keratin, metabolic disorders and hyperhidrosis [139].

### 5.2. Trimethylamine–Fishy Smell

In patients with TMAu, excessive amounts of unmetabolized trimethylamine are also exuded from the skin surface (with sweat) causing characteristic fish-like body odor, which can be noticed regardless of patient’s good personal hygiene [50,143]. In a study by Wise et al. [53] on patients with idiopathic malodor approximately one third tested positive for trimethylaminuria. Among these individuals self-identified body odor was a chief symptom (29.9%) followed by body and oral malodor combined (21.4%). However, only 5% of TMAu-positive patients indeed had noticeable malodor being detected on the palms of the hand and none of them emitted body malodor noticeable at social distances. After the consumption of choline (which is a substrate in synthesis of TMA) as much as 10% of enrolled subjects had noticeable body odor at social distances.

### 5.3. Ammonia–Urine-Like Smell

It has been shown that eccrine sweat contains ammonia [124,144]. However, the origin of NH_3_ in sweat has not been confirmed. Some researchers concluded that it is transported from plasma [145] while others claim that it comes directly from sweat glands [75]. The results of studies that aimed to compare concentrations of ammonia in breath and in sweat are also inconclusive. Furukawa et al. [73] showed that the dermal emission of odorous NH_3_ was higher than exhalation with breath. On the other hand, ammonia was present at lower levels in skin gas than in breath of individuals that were enrolled in a study by Turner and colleagues [74].

### 5.4. Methionine and its Metabolites–Smell of Rancid Butter or Boiled Cabbage

In patients with methionine adenosyltransferase I/III deficiency (which results in hypermethioninemia) who have not been diagnosed in newborn screening, a specific odor resembling boiled cabbage or rancid butter might be noticeable not only in breath or urine, but also in sweat. This distinctive smell is most likely caused by the formation of odorous DMS from methionine [13,76].

### 5.5. 2-Nonenal–Greasy, Grassy Smell

Chemical composition, intensity and pleasantness of natural body smell changes during a lifetime. It is known that elderly people have a specific body odor, sometimes described as “nursing home smell” [83]. Changes in body odor that are associated with aging have been investigated by Haze et al. [84]. The researchers found a specific particle, 2-nonenal that is characteristic for body smell of elderly and middle-aged people. This unsaturated aldehyde with a distinctive greasy and grassy odor is produced by the degradation of ω-7 monounsaturated fatty acids in skin surface lipids. The results of a study by Kimura et al. [99] support these findings.

## 6. Reproductive Fluids

Normal vaginal secretions are almost odorless or have a smell similar to that of yogurt. Thus, cheesy or fishy odor released with reproductive fluids might be a symptom of infections located in the genital area (e.g., bacterial vaginosis, trichomoniasis or candidiasis) as well as noninfectious conditions such as urinary incontinence, malignant ulcers, trimethylaminuria or chronic constipation [146].

Bacterial vaginosis is the most common cause of vaginal malodor. Other symptoms include vaginal discharge, itching and irritation. Abnormal, fishy smell of vaginal fluids in this condition is caused by volatile amines (putrescine, cadaverine, TMA) produced by bacteria such as *Gardnerella vaginalis* [59]. In most cases, bacterial vaginosis can be quickly cured with antibiotics. However, in approximately one third of patients, regular treatment does not improve the symptoms.

Noninfectious causes for vaginal malodor are less common than infectious ones and thus they pose a greater clinical challenge. Physicians should also appreciate that odor perceived as vaginal might be of different origin such as anal canal or urinary tract. The unpleasant smell of genital sweat can also be mistaken for vaginal [140].

Gastrointestinal conditions, such as chronic constipation and fecal incontinence should also be recognized as possible causes for malodor emitted from anogenital area. Volatile odorous compounds in these states would include hydrogen sulfide, methanethiol and dimethyl sulfide, which are responsible for the odor of flatus and stool [47]. Vaginal examination in these patients will not show any abnormalities.

Finally, some patients with gynecological tumors and lesions complain of vaginal discharge with foul smell. For example, the malignant ulcers of the vulva with necrosis might result in offensive, rotting odor, presumably due to the formation of putrescine, cadaverine, short-chain fatty acids (isovaleric and butyric acids) and sulfur containing compounds by bacteria [56].

## 7. Management

Regrettably, evidence-based guidelines for the management of body malodor are lacking and no universal treatment exists. Several temporary solutions such as teeth brushing, mouthrinses, chewing gums, or frequent bathing with antibacterial soaps and using deodorants have been discussed in medical literature. However, these methods do not solve the underlying problem, but rather mask or reduce the unpleasant smell to an acceptable level [5,146]. A satisfying outcome can only be achieved when the causing factor is taken into consideration.

In general, malodor that is associated with accumulation of bacterial metabolites in body fluids results from imbalance between the synthesis and excretion of odorants. This might be caused by one or more of the following factors: (i) diet containing odorants or substrates for their production, (ii) “pro-odor” bacteria composition and metabolic activity of bacteria, (iii) increased absorption of odorants or their precursors from the gut (increased intestinal transit time, increased gut-blood barrier permeability), (iv) decreased liver metabolism of odorants and (v) decreased urinary excretion.

Based on the above, some general, but not evidence-based, preventive measures may be advised in addition to standard hygienic procedures.

1. Reduction of dietary substrates for odorant production.
2. Frequent bowel movements to reduce passage time (to shorten time of gut bacterial metabolism and time of absorption of bacterial metabolites), treatment of constipation.
3. Probiotic and prebiotic treatment (an attempt to modify gut bacterial composition).
4. Increased water intake in order to increase excretion of metabolites with urine.

The following paragraphs address these measures based on the type of odorant. Table 3 summarizes therapeutic options for malodor associated with bacterial odorants.

Management by the type of odorant:

### 7.1. Hydrogen Sulfide

Since hydrogen sulfide mostly contributes to the bad smell of breath in intra-oral halitosis management should be focused on dental hygiene and treatment of periodontal disease [5].

Recently Suzuki et al. [147] confirmed that zinc ions might be useful in inhibiting oral malodor caused by excess of gaseous hydrogen sulfide. Two mechanisms were responsible for the effect of these ions—direct binding of H_2_S by Zn^2+^ and suppression of growth of oral bacteria. Another promising solution might be the use of mouthwash with chlorine dioxide. It has been reported useful in reduction of morning oral malodor, dental plaque and tongue coating. It also reduces count of *Fusobacterium nucleatum* in saliva [148]. However, long-term effects of these solutions remain unknown.

Finally, changes in diet may be beneficial. Restriction of products containing sulfides and sulfites which are the substrates for the production of H_2_S by bacteria should be advised. This includes avoidance of onion, garlic, wine, cabbage, broccoli, mushrooms, nuts, potatoes and dried fruits which are often preserved with sulfur dioxide.

### 7.2. Methanethiol

Methanethiol is a predominant substance that is involved in intra-oral halitosis. Similarly to management of high hydrogen sulfide content in breath, taking into account that some bacterial species (e.g., *Prevotella*, *Veillonella*, *Atopobium*, *Megasphaera*, and *Selenomonas* [19]) are able to produce this molecule, researchers raise the importance of tongue coating reduction and treatment of periodontal disease in patients with bad breath.

The presence of odorous methanethiol in urine is often associated with consumption of asparagus [48]. Thus, association between occurrence of unpleasant smell and asparagus ingestion should be considered before further diagnosis and potential treatment.

### 7.3. Trimethylamine

It needs to be pointed out that no single therapy regimen has been found to be universally efficacious in systematic studies. Nonetheless, treatment should be focused on the elimination of accumulated TMA from circulation. As TMA is produced by gut bacteria from diet-derived substances such as choline, betaine and L-carnitine, decreasing their intake by limiting red meat [149,150] egg yolks or soybeans [151] in diet may be effective [152]. Since gut bacteria may also convert TMAO to TMA, the reduction of products containing TMAO (fish, seafood) might be advised. Finally, the use of probiotics (to reduce the amount of TMA-producing bacteria) or activated charcoal that can bind TMA in the gut [88] might come in useful.

As for management of bad smell of sweat, along with other therapies that were discussed above, the use of low pH soaps and antiperspirants has been reported effective in reducing body odor [153].

### 7.4. Indole and Skatole

Management should include treatment of periodontal disease, as significant amount of these compounds comes from metabolism of tryptophan by anaerobic bacteria [28]. Additionally, decreasing tryptophan intake (to minimal recommended doses) might help to reduce malodor that is caused by indole and skatole. This could be done by reducing daily consumption of oats, bananas, milk, tuna, cheese, bread, poultry, nuts, seeds, and chocolate [154,155].

### 7.5. Putrescine and Cadaverine

Putrescine and cadaverine–diamines detectable in breath of some patients with halitosis have low volatility in neutral pH of oral cavity and are not a major component of foul smell [156]. Simple teeth hygiene and food consumption (mechanical action of chewing food) can significantly decrease the levels of these diamines in saliva, according to Cooke et al. [121].

### 7.6. Pyridine

Research on the role of pyridine in body malodor is very limited. However, it has been detected in the breath of patients with periodontal disease and subsequent halitosis. Therefore, the treatment of periodontal disease and oral hygiene should be recommended [33].

### 7.7. Ammonia

First, the detection of ammonia’s smell requires exclusion of life-threatening conditions such as liver failure. Its presence in exhaled air mostly comes from the blood. Hence, elimination of NH_3_ from the blood will also decrease breath ammonia (as well as its levels in other body sites). As ammonia is produced during metabolism of proteins, high-protein diets can increase the breath ammonia levels [133]. Thus, reasonable protein content in diet might also help keeping breath ammonia in a normal range.

Current treatment of hyperammonemia includes rifaximin (nonsystemic, GI-site specific antibiotic), which minimizes intestinal production of NH_3_ by bacteria and lactulose, which decreases absorption of gut-derived NH_3_ into the blood [129]. Furthermore, l-Ornithine l-Aspartate combination has been lately reported efficacious in lowering ammonia blood levels in patients with hepatic encephalopathy [157].

Finally, some amount of ammonia in breath comes from bacterial hydrolysis of urea present in the saliva. Thus, the removal of tongue coating and dental plaque can help to reduce bad smell in intra-oral halitosis [71].

### 7.8. (E)-3-Methyl-2-Hexenoic Acid (E3M2H), (R)/(S)-3-Hydroxy-3-Methylhexanoic Acid ((R)/(S)-HMHA) and 3-Methyl-3-Sulfanylhexan-1-ol ((R)/(S)-MSH)

As for treatment of bromhidrosis, improving hygiene along with the use of antiperspirants and topical antibacterial agents is indicated. The reduction of bacterial count has been found to improve malodor in patients with axillary and plantar osmidrosis [158]. In more severe cases, laser subdermal coagulation [139] or surgical methods [159,160,161,162] might be helpful. The use of BTX-A (botulinum toxin type A) has also been reported to be effective in reducing odor in patients with bromhidrosis [163].

Finally, in eccrine bromhidrosis, which can be exacerbated by consumption of garlic, curry, onion, and alcohol; the avoidance of these products can be helpful [139].

## 8. Conclusions

To date several compounds that are responsible for body odor have been identified and most of them are of bacterial origin. Although no evidence-based guidelines for management of body malodor exist, some therapeutic measures targeting diet, microbiota composition and personal hygiene may alleviate the symptoms. Increased awareness of physicians and further research is needed to address the problem of malodor in otherwise healthy patients. This is of high importance as malodor might lead to serious psychological problems for both patients and their families.

## 9. Materials and Methods

We searched PUBMED and Google Scholar databases to identify clinical and pre-clinical studies on unpleasant body smell. The key words included microbiota, dysbiosis, halitosis, body odor, malodor, bad breath, and bad smell. The search was confined to manuscripts that were published in English before February 2019.

## Figures and Tables

**Table 1:** Most common bacterial odorants.

Substance	Smell	Body Site	References
Hydrogen sulfide	Rotten eggs	Breath, Saliva, Flatus, Urine	[18,19,44,45]
Other volatile sulfur compounds (VSCs): ethanethiol, S-ethyl thioacetate, diethyl disulfide, dimethylsufide	Cooked onion or vegetables, ocean; musty, unpleasantly sweet smell	Breath, Saliva	[20,23,26]
Methanethiol	Putrid, barnyard; musty smell	Breath, Saliva, Flatus, Urine	[20,26,46,47,48]
Trimethylamine	Rotten fish	Breath, Urine, Sweat	[38,49,50,51,52,53]
Indole, Skatole	Fecal matter	Breath, Saliva	[28,30]
Short chain fatty acids (butyric, propionic, acetic, isovaleric, isocaproic acid)	Human vomit, sweat, goat-like, sweaty feet odor; cheesy smell	Sweat, Urine, Vaginal discharge	[54,55,56]
Putrescine, Cadaverine	Rotten meat, spoiled fish	Breath, Saliva, Vaginal fluid (in bacterial vaginosis)	[32,57,58,59]
Acetone	Acetone, fruity smell of rotten apple	Breath	[60,61,62]
Pyridine	Fishy odor	Breath, Saliva	[63,64]
(E)-3-methyl-2-hexenoic acid (E3M2H)	Peculiar pungent odor	Sweat	[65,66,67]
3-methyl-3-sulfanylhexan-1-ol ((R)/(S)-MSH)	Tropical fruit or grapefruit (enantiomer R), onion, clary sage, chicken-sulfury (enantiomer S)	Sweat	[65]
(R)/(S)-3-hydroxy-3-methylhexanoic acid ((R)/(S)-HMHA	Cheesy, rancid odor	Sweat	[68,69,70]
Ammonia	Urine-like, ammoniacal, fetid	Breath, Saliva, Urine, Stool, Sweat	[34,71,72,73,74,75]
Methionine (transformed into dimethylsulfide)	Boiled cabbage, rancid butter, oast house, rotten mushrooms	Breath, Sweat, Urine	[11,12,76]
Phenylacetate	Musty, mousy, sweaty	Urine, Infant skin	[77]
Branched-chain amino acids (leucine, isoleucine and valine)	Maple syrup, caramelized/burnt sugar, fenugreek, curry	Urine, Ear wax	[78,79]
3-hydroxyisovaleric acid	Male cat urine	Urine	[80,81]
Aldehydes (2-nonenal)	Foul, urine-like	Urine, Skin	[82,83,84]

**Table 2:** Conditions associated with malodor.

Condition	Smell	Body Site	References
Intra-oral halitosis	Rotten eggs, cooked onion or vegetables, putrid, musty, urine-like smell, fecal matter, rotten meat or fish	Breath, Saliva	[8,21,27,32,85]
Trimethylaminuria	Rotten fish	Breath, Urine, Sweat	[39,49,86,87,88]
Phenylketonuria	Musty, mousy, sweaty smell	Urine, Infant skin	[77,86,89]
Maple syrup urine disease	Maple syrup, caramelized/burnt sugar, fenugreek, curry	Urine, Ear wax	[78,79,86,90]
Diabetic ketoacidosis	Acetone, fruity smell of rotten apple	Breath	[9,10,62]
End-stage renal disease	Urine-like smell, ammoniacal, rotten fish	Breath	[35,91,92]
Liver failure	Urine-like smell, ammoniacal, rotten fish	Breath	[38,93,94]
Urinary incontinence	Rotten eggs, putrid, musty, urine-like smell	Urine	[72,82,95]
Methionine adenosyltransferase (MAT) I/III deficiency	Putrid, musty, smell of rancid butter or boiled cabbage	Breath, Urine, Sweat, Skin	[11,12,13,96]
Isovaleric acidemia	Human vomit, sweat, goat-like, sweaty feet odor; cheesy smell	Sweat, Urine	[54,55,86,97]
3-methylcrotonyl-CoA carboxylase (3-MCC) deficiency	Smell of male cat urine	Urine	[80,81]
Bromhidrosis	Rancid, cheesy smell of sweat	Sweat, Skin	[65,66,67,68,69,70,98]
Aging	Greasy, grassy smell	Sweat, Skin	[84,99]
Bacterial vaginosis	Fishy smell	Vaginal discharge	[59]
Gynecological lesions	Rotting smell	Vaginal discharge	[56]

**Table 3:** Management by the type of bacterial odorant.

Bacterial Odorant	Conditions	Management	References
Hydrogen sulfide	Intra-oral halitosis (periodontic disease, excessive tongue coating), UI and UTIs	Basic dental hygiene, treatment of periodontal disease, mouthwashes with chlorine dioxide or Zn^2+^ ions; avoidance of sulfites and sulfides in diet	[5,101,102,103,147,148]
Methanethiol	Intra-oral halitosis (periodontic disease, excessive tongue coating), treatment with cysteamine, hepatic methionine adenosyltransferase deficiency, UI	Basic dental hygiene, treatment of periodontal disease, avoidance of asparagus in diet	[19,23,48]
Trimethylamine	Trimethylaminuria, bacterial vaginosis, liver failure, ESRD	Avoidance of products containing choline, betaine, l-carnitine or TMAO in diet; administration of probiotics and activated charcoal; use of low pH soaps and deodorants	[88,149,150,151,152,153]
Indole, Skatole	Intra-oral halitosis	Decreasing tryptophan intake to allowed minimum; treatment of periodontal disease	[28,154,155]
Putrescine, Cadaverine	Intra-oral halitosis, bacterial vaginosis, gynecological lesions	Basic dental hygiene, antibiotics for bacterial vaginosis, treatment of underlying gynecological problems	[121]
Pyridine	Intra-oral halitosis (periodontic disease)	Basic dental hygiene, treatment of periodontal disease	[33]
Ammonia	Intra-oral halitosis (periodontic disease, excessive tongue coating), extra-oral halitosis, liver failure, ESRD, UI, bromhidrosis	Avoidance of high-protein diets; l-Ornithine l-Aspartate, rifaximin, lactulose; basic dental hygiene, treatment of periodontal disease	[71,129,133,157]
E3M2H, (R)/(S)-HMHA, R)/(S)-MSH	Bromhidrosis	Personal hygiene with the use of antiperspirants and topical antibacterial agents; avoidance of garlic, onion, alcohol and curry in diet; subdermal coagulation, surgical methods, BTX-A	[139,158,159,160,161,162,163]
